# Coil Catastrophe Averted: A Technical Note on Retrieval From the Basilar Artery

**DOI:** 10.7759/cureus.104323

**Published:** 2026-02-26

**Authors:** Nicholas Es'haghi, Yasir Khattak, Muhammad Manzoor, Ramon Navarro

**Affiliations:** 1 Radiology, Townsville University Hospital, Townsville, AUS; 2 Interventional Neuroradiology, Townsville University Hospital, Townsville , AUS; 3 Neurological Surgery, Townsville University Hospital, Townsville , AUS

**Keywords:** aneurysm, endovascular, migration, radiology interventional, retrieval

## Abstract

Early treatment of vertebral artery dissecting aneurysms (VADA) is associated with improved outcomes. Endovascular treatment options utilising both deconstructive and reconstructive techniques have been used. Coil migration may occur during parent vessel occlusion as a rare complication that can lead to further thromboembolic events. Various strategies for coil retrieval have been documented; however, management currently relies on the discretion of the proceduralist, as there is no established consensus on a standardised salvage technique.

This technical note describes a coil embolisation parent artery occlusion for a ruptured VADA complicated by a migrated coil into the basilar artery and a salvage technique using a stent retriever and aspiration catheter.

The coil mass was successfully retrieved from the basilar artery using a hybrid-cell stent retriever and a larger-bore aspiration catheter. The aneurysm was then treated with parent vessel reconstruction using a flow diverter stent. Post-operatively, ischaemic changes were noted in the left temporal lobe. At the six-month follow-up, she has a modified Rankin Scale (mRS) score of 2 and is currently participating in rehabilitation.

Coil migration during endovascular parent artery sacrifice is a rare complication. In this technical note, we describe how using a hybrid-cell design stent retriever in combination with a large-bore aspiration catheter, positioned across the long axis of the coil mass, enabled successful retrieval in a single VADA case.

## Introduction

Intra-dural vertebral artery dissecting aneurysms are a well-known cause of subarachnoid haemorrhage. Early treatment of such aneurysms is warranted to prevent rebleeding [1,2]. Several endovascular techniques, including parent vessel occlusion (deconstructive) and flow-diverter stent placement (reconstructive), are widely utilized for the management of dissecting intracranial aneurysms. Parent vessel occlusion is the deliberate sacrifice of an affected blood vessel to stop flow through a pathological segment, whilst a flow diverter stent preserves the parent vessel by placing a stent across the diseased segment, redirecting blood away from the abnormal area. Coil migration is a recognised, although rare, complication of vessel sacrifices [3,4]. It is of particular concern in high-flow vessels when proximal flow can push the coil mass [4]. Other factors contributing towards coil migration include the size and length of the coils, location, target geometry and the technique [1,5]. Coil migration can impede distal flow and often warrants coil retrieval to salvage and restore distal flow, especially in cases of basilar apex migration, where several critical vessels can be partially or completely obstructed. Currently, there is no consensus salvage technique for coil retrieval, with several methods described in the literature using retrieval devices, snares, aspirators, alligator clips and wire techniques [3,5,6]. Furthermore, there are limited reports on the deployment strategy and approach in this patient cohort.

We present a case of a right vertebral artery (V4) ruptured dissecting aneurysm, initially treated with parent vessel occlusion, which was complicated by a migrated coil mass into the basilar artery. The coil mass was successfully retrieved using a combination of a hybrid-cell stent retriever and a large-bore aspiration catheter optimally positioned across the long axis of the coil mass. Subsequently, the dissecting aneurysm was treated with parent vessel reconstruction using a flow-diverter stent. In this technical note, we propose our recommendations for a hybrid stent-retriever and long-axis deployment to maximise coil retrieval.

## Technical report

A 52-year-old female presented to our hospital with an atraumatic, sudden-onset headache and confusion - World Federation of Neurosurgical Societies Grade II. Initial computed tomography (CT) of the head and neck demonstrated intraventricular haemorrhage, and an acute large volume diffuse subarachnoid haemorrhage (SAH; modified Fischer Grade IV) concentrated in the basal cisterns. CT angiogram revealed a left transitional/carotid cave internal carotid artery (ICA) aneurysm arising from its medial aspect measuring approximately 3 x 2 mm; as well as a subtle linear hypodensity in the right intradural vertebral artery (V4) extending to the proximal basilar artery, suspicious for a dissecting aneurysm. The patient underwent diagnostic digital subtraction angiography (DSA), which confirmed the presence of a 6 x 3 mm right vertebral artery fusiform dissecting aneurysm proximal to the origin of the posterior inferior cerebellar artery (PICA). The diameter of the vertebral artery proximal and distal to the aneurysm measured 2.6 mm and 2.2 mm, respectively. Considering the acute, ruptured aneurysm, our initial decision was made to proceed with proximal vessel sacrifice rather than a reconstructive technique to avoid the use of antiplatelets in the acute setting. 

**Figure 1:**
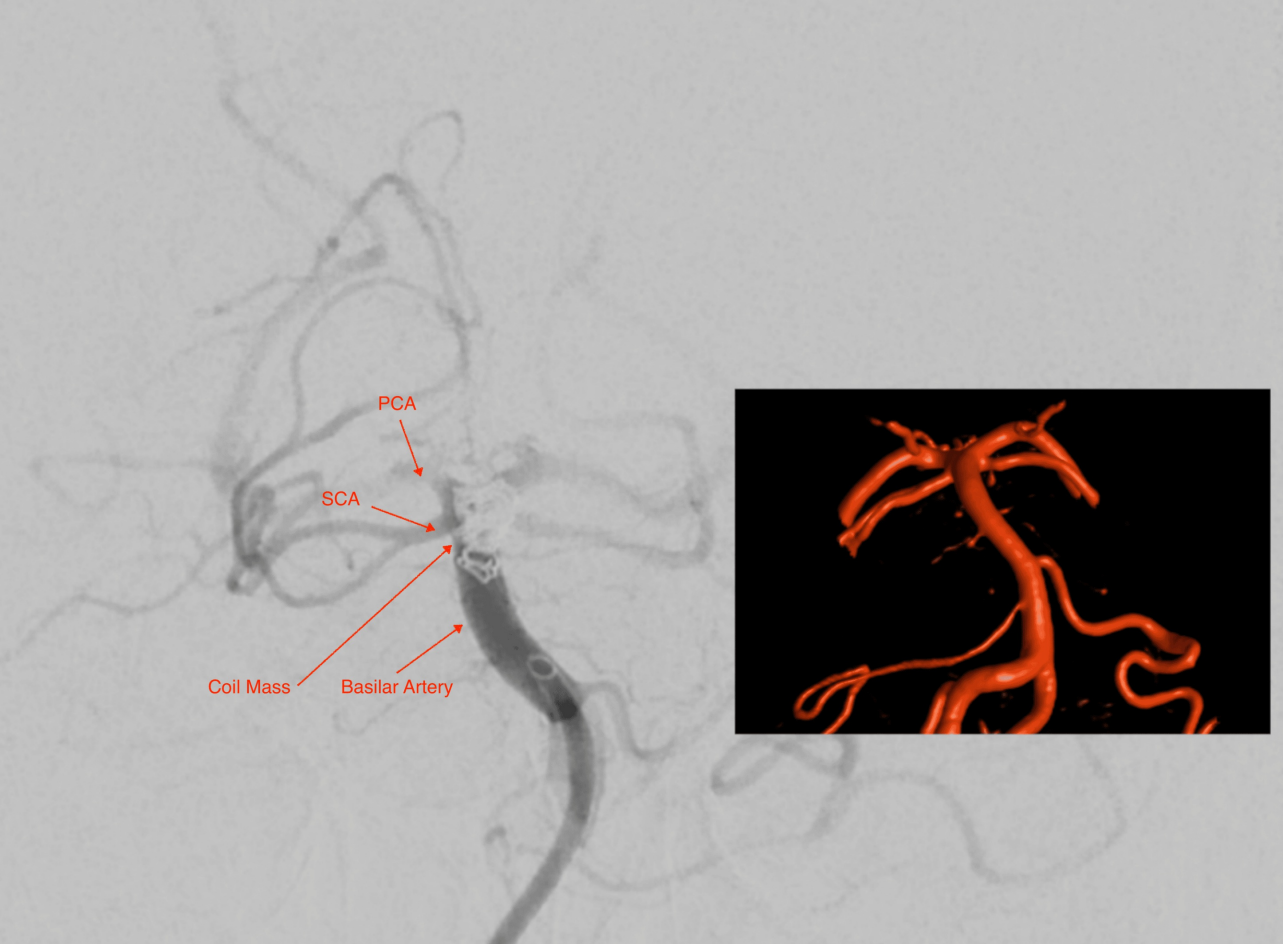
Intra-operative imaging of the final migrated coil mass position and its association with the basilar artery, superior cerebellar artery (SCA) and posterior cerebral artery (PCA). Overlayed 3-dimensional reconstructed CT helps visualise the anatomy.

Access was secured in the right vertebral artery using a 6 Fr guiding sheath, and a 6 Fr intermediate catheter was placed in the V3 segment of the right vertebral artery. Subsequently, an SL-10 microcatheter (Stryker, Michigan, USA) was used to deliver five coils (a combination of 1 (4 mm x 8 cm) HydroFrame 10 (Terumo, Tokyo, Japan), 2 (3 mm x 6 cm) HydroCoil 10 (Terumo, Tokyo, Japan), one Hydrosoft 3D (Terumo, Tokyo, Japan) and two Target Tetra (Stryker, Michigan, USA) 2.5 mm x 6 cm) inside the aneurysm extending slightly proximal to it. During the final runs with the guiding catheter in a more proximal position, the coil mass began to migrate distally and ultimately reached the basilar artery apex (Figure 1). Access to the contralateral vertebral artery was obtained to avoid manipulation of the dissecting aneurysm during coil retrieval manoeuvres. A combination of different microcatheters, stent retrievers, and aspiration catheters had to be used to remove the coil mass (Video 1). The first retrieval attempt was made using a closed-cell Solitaire stent (Medtronic, Minnesota, USA) (4 x 20 mm) through a Sofia 5F (Terumo, Tokyo, Japan) catheter for aspiration. The Solitaire stent was passed along the short axis of the coil mass towards the right posterior cerebral artery, since access to the left one was partially blocked by the coil mass and deployed with unsuccessful retrieval. We hypothesize these technical factors led to insufficient wall apposition, contact area and stent integration contributing to failure. This approach was abandoned, and the second attempt utilised a slightly longer stent retriever with a hybrid cell structure (NeVa (4mm x 22mm) Vesalio, Texas, USA), which was deployed again from the right posterior cerebral artery to the basilar trunk. However, this was also unsuccessful in capturing the coil mass, presumably secondary to short-axis positioning and suboptimal wall apposition. In the third attempt, we decided to try to cross the coil mass and gain access to the left posterior cerebral artery. Retrieval was finally achieved with a NeVa (4 mm x 22 mm) from the left posterior cerebral artery and a Vecta 74 (Stryker, Michigan, USA) aspiration catheter to pinch the coil mass. In this attempt, the NeVa was passed across the long axis of the mass, allowing for greater interaction between the stent and the coils. The coil mass got partially dislodged at the proximal V4 segment, and the remaining coils could be directly aspirated using the Vecta 74. Angiographic runs after removal of the coil mass did not show any filling defects in the posterior circulation.

**Video 1:** Technical video of procedure.

We opted to pivot our approach, and subsequently, the aneurysm was repaired using a reconstructive strategy to avoid the risk of having a second coil migration event. The implications of this requiring antiplatelet therapy in the acute SAH setting were considered, at the time an external ventricular drain was already in situ, and no further surgical interventions were anticipated, which made us more comfortable to use dual-antiplatelet therapy. Therefore, we proceeded with intravenous loading of 300 mg aspirin and infusion of tirofiban. A pipeline vantage (3.5 x 16 mm) (Medtronic, Minnesota, USA) flow diverter stent was deployed in the parent vessel across the dissecting segment. Adequate coverage of the dissecting aneurysm and good wall apposition were confirmed with a post deployment DynaCT (Siemens Healthineers, Erlangen, Germany) with diluted contrast. Total fluoroscopic procedure time was one hour and seven minutes.

Although no major vessel occlusion occurred, ischemic changes were noted in the left temporal lobe. We are pleased to report that the patient is making a favourable clinical recovery; at the six-month follow-up, she has a modified Rankin Scale (mRS) score of 2 and is currently participating in rehabilitation.

## Discussion

Migration of a coil during endovascular treatment of intracranial aneurysms is a recognised complication with potentially serious consequences if not appropriately managed [7]. Reported rates of migration range from 1.7% to 6% [7]. Most cases occur while deploying the final coil; however, delayed postprocedural migration has also been noted [8]. Aneurysms most susceptible to coil displacement are small, communicating segment aneurysms and those with a wide neck, typically having a low aspect and dome-to-neck ratio less than 2:1 [1,5]. Technical risk factors have also been described, including smaller coils, mismatch of coil to aneurysm size and deployment or retrieval of the first or last coil [5,8,9]. 

Pre-embolisation vessel and aneurysm size evaluation plays a fundamental role in reducing the risk of coil migration [9,10]. A thorough assessment includes both the anatomical location of the target vessel and haemodynamic parameters, such as blood flow velocity and volume. These factors have a direct influence on the stability of the embolic construct. Coil size selection is equally important. Various studies favour that deliberate oversizing reduces the likelihood of coil migration by improving anchorage within the vessel lumen. In our case, there was no major discrepancy between the dissecting aneurysm diameter and the parent vessel, reducing coil anchoring and stability despite oversizing the coils approximately 15% to the aneurysm maximum diameter (4 mm coil for 3.4 mm) and 45% relative to the distal vessel diameter (4 mm coil for 2.2 mm).

In the technical video referenced, coil migration was observed after complete deployment and during the final phase of the procedure. This complication may have been prevented by creating a more robust coil mass through the use of an initial wider coil, longer coils, or by selecting coils specifically engineered for permanent vessel occlusion, such as POD coils (Penumbra Inc., California, USA) or Optiblock coils (Balt Group, Montmorency, France) [11]. Alternatively, adjunctive devices, including microvascular plugs (e.g., MVP™ system, Medtronic, Minnesota, USA), can provide additional mechanical stability when placed proximal to the coil mass and reduce the risk of displacement [12].

It is of paramount importance to be mindful of the haemodynamic alterations resulting from larger-calibre intermediate catheters, which can significantly reduce the flow pressure in the parent vessel. Thus, understanding that during coiling and subsequent intracranial angiographic runs performed to confirm stability, operators should avoid techniques that excessively obstruct the vessels [4]. Maintaining near-physiological haemodynamic conditions during these assessments ensures that the behaviour of the coil mass accurately reflects real-time flow dynamics, thereby allowing more reliable prediction of long-term stability. Our guiding catheter position during coiling could have been too obstructive, and a slight pull on it to perform the final runs likely increased the flow and prompted coil migration.

Management varies depending on timing, location, length of migration, target vessel patency, and eloquence of the vascular territory [3,7]. Minor coil protrusions from aneurysms are suspected to reendothelialise and become incorporated into the vessel wall; thus, they can be conservatively treated with 24-48 hours of anticoagulation followed by 6 months of antiplatelet therapy [3]. Instances where coil retrieval should be considered are those when a significant migration occurs intraoperatively, migration to a proximal or eloquent vessel and/or associated occlusion [8]. The proceduralist must weigh up the risks of resultant thromboembolism and/or haemorrhage if coil retrieval is attempted with the benefits [3,7]. Two endovascular broad salvage techniques have been described -coil mass retrieval or fixation of the coil against the vessel wall with a stent [7]. Retrieval is often favoured given the potential thrombogenic risks associated with a second implant and the required dual antiplatelet therapy [3,7]. In addition, when the coil mass is substantial, the chances of securing the coils against the wall and maintaining the vessel flow are slim.

**Figure 2:**
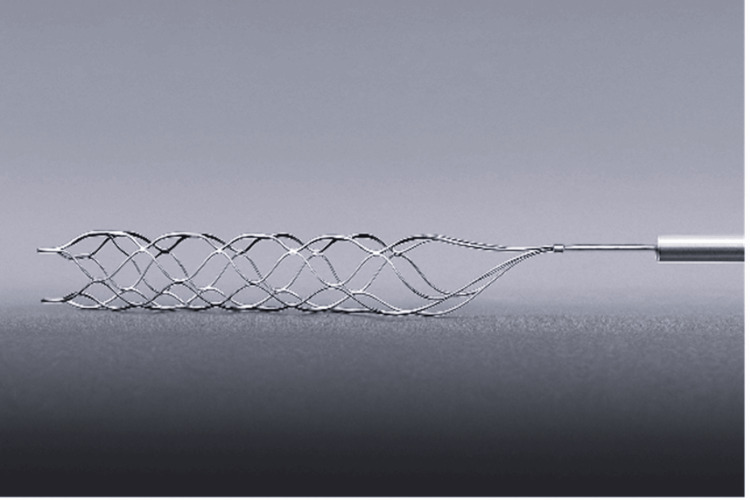
Solitaire™ X revascularization device. Example of a closed-cell device.

**Figure 3:**
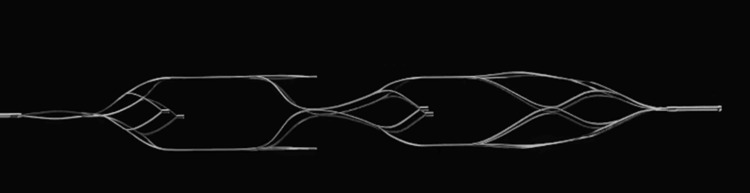
NeVa Stent retriever with multiple drop zones. An example of a hybrid-stent retriever with a open-cell and closed-cell areas.

Currently, no standardised coil retrieval techniques exist; however, several reports describe various strategies utilising snare devices, retriever devices and open microsurgical removal [3,7,13]. The use of stent retrievers in coil retrieval has become increasingly common and poses several advantages over other techniques, including: familiar use in mechanical thrombectomy, multiple contact points, structural integrity, and contact area [13]. The first successful retrieval of a dislocated coil using a stent retriever was published in 2010, with subsequent cases published utilising similar closed-cell devices [5,13]. There remains a paucity of literature on the superior retriever design and technique for coil retrieval. Stents can be classified based on the strut structure into closed-cell (Figure 2 - small free cells between struts) and open-cell (large, uncovered gaps) [14]. Hybrid designs also exist, combining open cells in the proximal and distal ends with a central closed-cell area (Figure 3). These subtle technical considerations might increase the success rate of this rescue procedure, thereby reducing fluoroscopic time and intravascular artery manipulation and chances of embolic complications.

**Figure 4:**
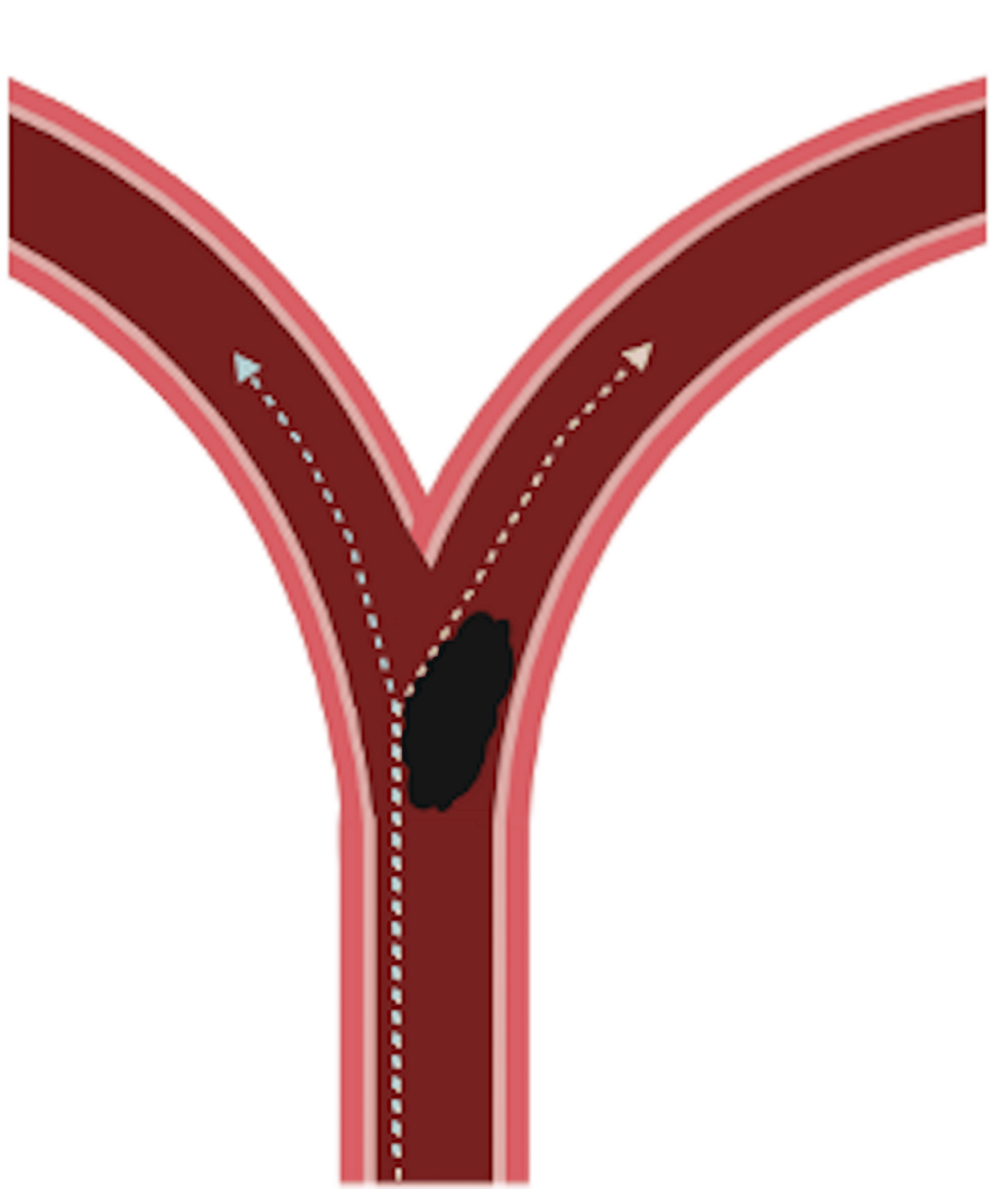
Authors illustrative diagram proposing the difference in contact area between stent deployment through the right vs left posterior cerebral artery. Authors original illustrative artwork.

**Figure 5:**
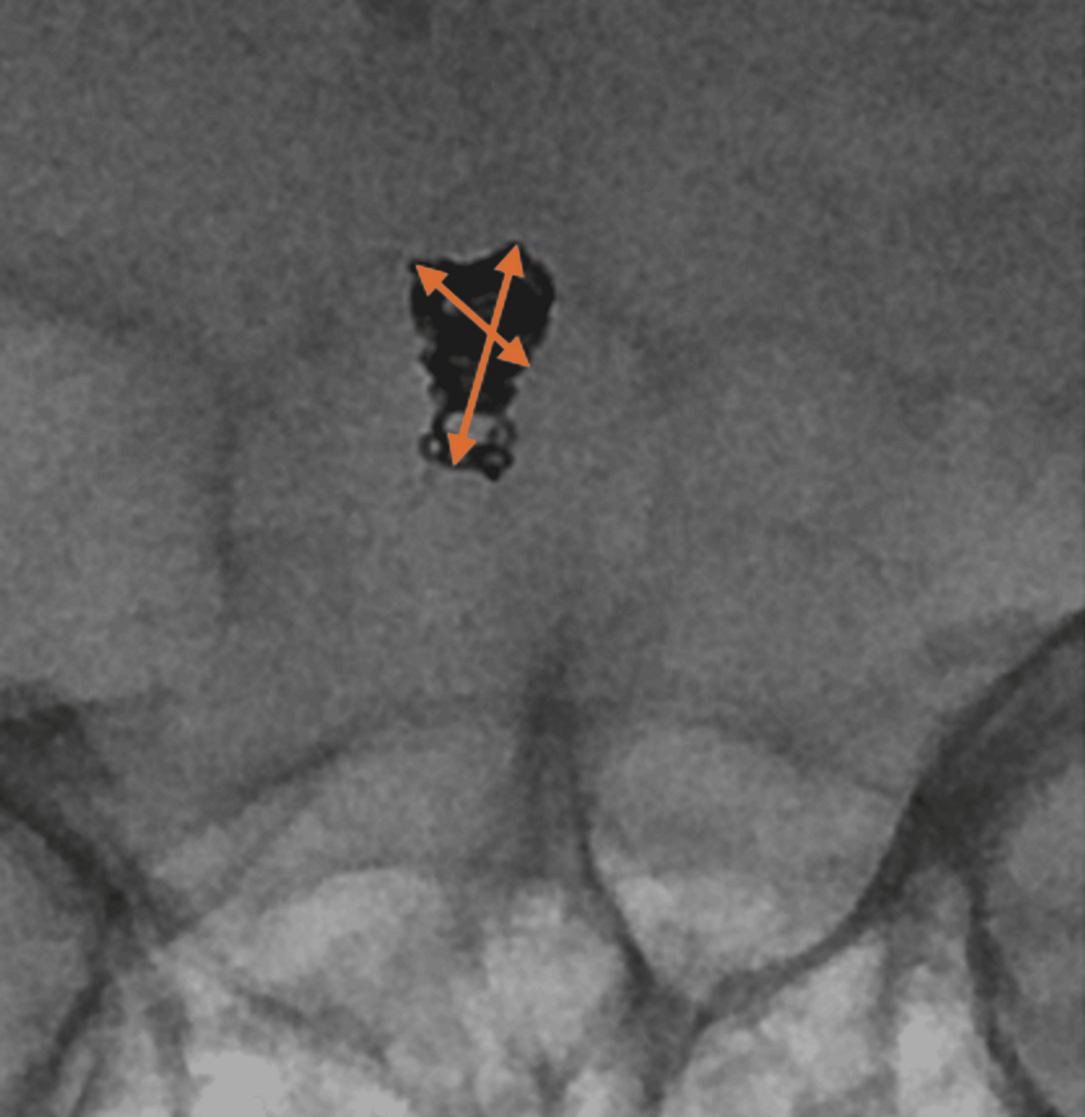
Intra-operative imaging of the final migrated coil mass position, highlighting the asymmetrical geometry with a short and long axis.

**Figure 6:**
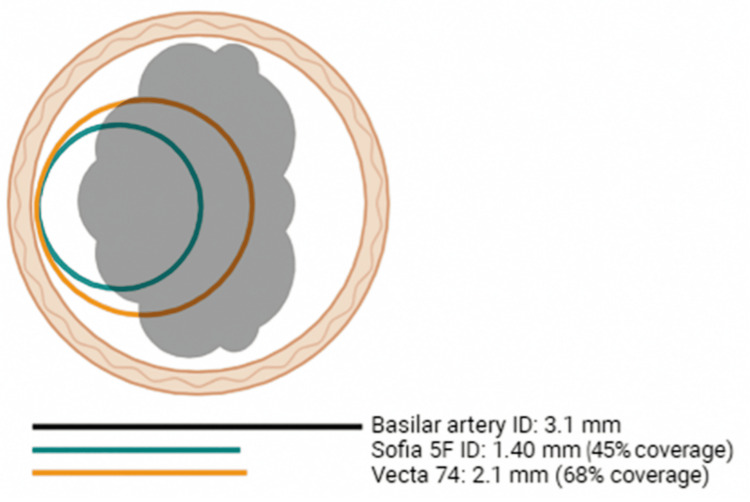
Schematic diagram of the different aspiration catheter diameters and their theoretical coverage and contact area with the coil mass. Author's original illustrative artwork - not quantitatively validated.

The conventional use of stent retrievers is to pin the clot against the arterial wall and withdraw it back [15]. The same paradigm is applied to a coil mass. However, the mechanical properties of the coils differ from those in a clot; the stent struts might cut through the clot and incorporate it into the stent, whereas the coils are not penetrable and are more likely to be displaced rather than incorporated in the stent. Based on our experience in this report, maximising contact between the retriever and the mass against the wall is crucial. Given the position of the coil at the bifurcation of the basilar artery, either side could be approached for stent positioning (Figure 4). Selecting the right posterior cerebral artery was easier since the coil mass did not completely block it; therefore, initial attempts were made with this configuration (stent from right posterior cerebral artery to basilar trunk). Initial retrieval was unsuccessful when passing the Solitaire X along the short axis of the mass (Figure 5). However, when a different design stent (NeVa) was passed along the long axis in the final attempt, a greater contact area and compression were achieved, allowing for the incorporation of the coils into the stent retriever (Figure 4). The NeVa stent was initially designed to capture hard clots by adding the so-called “drop zones” that can retain the bulk of the clot. Based on this experience, the use of a hybrid-cell stent system may be considered to maximise coil integration. Retrieval success may also be influenced by the use of a large-diameter aspiration catheter (Figure 6), which has been shown to be effective in aspiration thrombectomy for recanalisation of acute basilar artery occlusion [16,17]. In this case, the greater internal diameter of the Vecta 74 appeared to allow for greater contact and direct aspiration of the partially dislodged coils (Table 1). Where technically feasible, we would advocate for using a larger-bore catheter to achieve as much occlusion as possible proximal to the coil mass. It is essential to be aware that the area of the circumference increases significantly with its radius (A = πr²) to ensure a safe match between the catheter diameter and the vessel. We hypothesize that utilising these tools from the outset would likely have reduced ischemia time, vessel manipulation, and radiation exposure. Should this complication occur in the future, this hybrid approach would be our primary strategy.

**Table 1:** Aspiration catheters used during the procedure and their respective dimensions reported by the manufacturer.

Product	Type	Internal diameter	Distal outer diameter
Sofia 5F	Catheter	0.055 in (1.40 mm)	0.067 in (1.7 mm)
AXS Vecta 74	Catheter	0.074 in (1.88mm)	0.083 in (2.1 mm)

## Conclusions

Coil migration remains a potential complication during endovascular parent artery occlusion for treatment of VADA. In this case, the use of a stent retriever facilitated successful coil retrieval, reflecting similar techniques utilised for clot retrieval while leveraging its design. Conclusions are limited by the single-case nature of this report; our experience suggests that deploying a hybrid-cell stent retriever along the long axis of the coil mass, in conjunction with a large-bore catheter, may improve engagement of the migrated coils by increasing contact and integration within the stent. Further experience and research are required to investigate the optimal coil retrieval technique and establish consensus guidelines.
